# A Study on the Application of LSTM to Judge Bike Accidents for Inflating Wearable Airbags

**DOI:** 10.3390/s21196541

**Published:** 2021-09-30

**Authors:** So-Hyeon Jo, Joo Woo, Gi-Sig Byun, Baek-Soon Kwon, Jae-Hoon Jeong

**Affiliations:** 1 Department of Control and Instrumentation Engineering, Pukyong National University, Busan 48513, Korea; shjo960@naver.com (S.-H.J.); whj9419@naver.com (J.W.); 2 School of Mechanical System Engineering, Kunsan National University, Gunsan 54150, Korea; 3 School of IT, Information and Control Engineering Information and Control Engineering Major, Kunsan National University, Gunsan 54150, Korea

**Keywords:** wearable, artificial intelligence, human safety, bike accident, airbag, LSTM

## Abstract

The traffic accident occurrence rate is increasing relative to the increase in the number of people using personal mobility device (PM). This paper proposes an airbag system with a more efficient algorithm to decide the deployment of a wearable bike airbag in case of an accident. The existing wearable airbags are operated by judging the accident situations using the thresholds of sensors. However, in this case, the judgment accuracy can drop against various motions. This study used the long short-term memory (LSTM) model using the sensor values of the inertial measurement unit (IMU) as input values to judge accident occurrences, which obtains data in real time from the three acceleration-axis and three angular velocity-axis sensors on the driver motion states and judges whether or not an accident has occurred using the obtained data. The existing neural network (NN) or convolutional neural network (CNN) model judges only the input data. This study confirmed that this model has a higher judgment accuracy than the existing NN or CNN by giving strong points even in “past information” through LSTM by regarding the driver motion as time-series data.

## 1. Introduction

Due to personal mobility device (PM) diversification and the increased demand for delivery platforms, two-wheeled vehicles are quickly becoming popular. In particular, according to the decrease in outings and contacts since COVID-19, the demand for delivery has largely increased, in proportion to which small and large traffic accidents have increased, along with the increase in users of transportation means such as bicycles or motorcycles. According to the statistics of the Traffic Accident Analysis System (TAAS), a total of 38,453 bike accidents including PM happened in 2019 in Korea. Since bikes, unlike cars, do not have an enclosure to protect riders in the event of a traffic accident, a collision can cause direct impacts on the riders and separate the riders from the bike. In this case, the rider can collide with other objects after being separated from the bike, resulting in significant hits to the rider’s head and neck, resulting in serious injuries or deaths [1,2]. Although there are helmets, suits, and airbags as protective equipment to protect the riders, most of the riders, except for professional riders, usually wear only helmets in daily life. Additionally, there are many riders who ride bikes without even helmets.

Several methods for judging a person’s movement are being studied. Using an image through the camera, the movement of the arms and legs can be tracked or the facial expressions can be identified [3,4]. It is also possible to judge movement with a sensor rather than an image or video. The acceleration sensor, the angular velocity sensor, the height sensor, and the EMG sensor are used to measure the current movement, running, walking, and climbing stairs [5,6,7,8,9,10]. Based on these studies, research on motion measurement using artificial intelligence is also being conducted, which greatly contributes to the development of the healthcare and game industry [11,12,13,14,15,16,17,18,19,20]. Research related to motorcycle accidents is also increasing. A study by [21] provided an analysis of the accident sequence in the event of a two-wheeled vehicle accident. Another study [22] presented a magnetic, angular rate, and gravity sensor-based system that detects accidents with 95.2% accuracy through principal component analysis (PCA) and support vector machines (SVMs) and proposed a system that can be notified to emergency medical centers. In [23], the authors presented an idea for an efficient design method of a detection system that effectively uses a large number of sensors through a two-step detection algorithm based on the self-organizing maps (SOMs) method. The authors of [24] proposed a system that attaches inertial measurement devices to the driver’s head, torso, and rear of the motorcycle to determine the occurrence of an accident through a maximum a posteriori(MAP) classifier and transmits the driver’s location and accident information. Another study [25] provided data on airbags for jackets.

This paper proposes the LSTM as an artificial intelligence model used in airbag systems to protect riders in cases of accidents. Artificial intelligence can continuously improve accuracy through consistent data collection and learning, receiving much concern recently as a core technology in various industries. In the case of airbags, conventional products often inflate the airbag through a mechanical method or use the threshold value of a sensor, and 2–3 types of sensors are used together. Studies are underway to find the possibility of using artificial neural networks, instead of the threshold method used in the existing bike airbags, to produce an airbag system with high accuracy [26,27,28,29]. The precedent studies using other artificial intelligence models made judgments only with respect to the “incoming input data” and outputted the results. By contrast, in this paper, the proposed LSTM can use new and old data to judge the accident situation. The wearer’s state of motion depending on accident situations can be assessed to be a state of motion, with the order depending on time. It was supposed to judge between the accident situation and the non-accident situation by analyzing the currently incoming data while bringing the information on the past motion state together.

In this paper, Section 2, “Method and Design of the Proposed System”, introduces “Data collection and System Design” (Section 2.1) and “LSTM theory and design” (Section 3.1). Section 2.1 explains the method of data collection and the analysis and treatment method of the collected data to teach and test the artificial neural networks and introduces the hardware system for the corresponding system. Section 3.1 describes the design of the artificial neural network LSTM and the LSTM model used in this study. Section 3, “LSTM Experiment Results and Comparisons”, explains the simulations using test data, and the experiments using a testbed to confirm the operation of the actual airbag system. Through the simulations with test data, in particular, the performance differences between each artificial neural network NN, CNN, and LSTM are discussed. Section 4, “Conclusion”, analyzes the experiment results and concludes the study.

## 2. Method and Design of the Proposed System

### 2.1. Data Collection and System Design

To train and build the artificial intelligence model, fundamental data on the objects are necessary. The system under study was the operation of wearable airbags amid the occurrence of bike accident situations, for which it is necessary to distinguish whether the wearer’s motion state is a general motion state or a state in which an external force is applied to its body due to the occurrence of an accident. The collision types in terms of bike accidents are largely classified into the following five types:

1. Front collision;
2. Rear collision;
3. Left collision;
4. Right collision;
5. Fall, Roll, etc.

**Figure 1:**
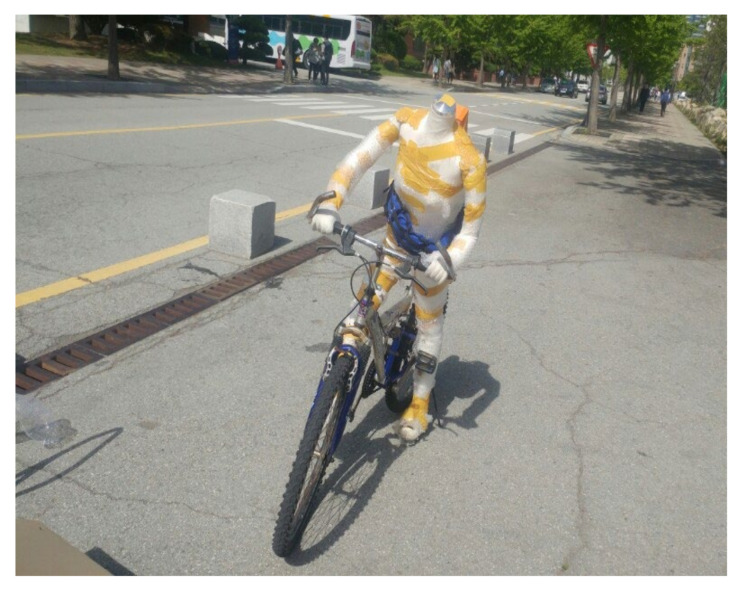
Mannequin and bike.

This study collected the accident data on the above items to be used as data on the accident situations and explains the analysis of the cases depending on the situations. However, because it is very dangerous for people to directly ride a bike and make an accident, a mannequin was used in place of people, as shown in Figure 1 [26,27,28,29].

#### 2.1.1. Raw Data Collection

**Figure 2:**
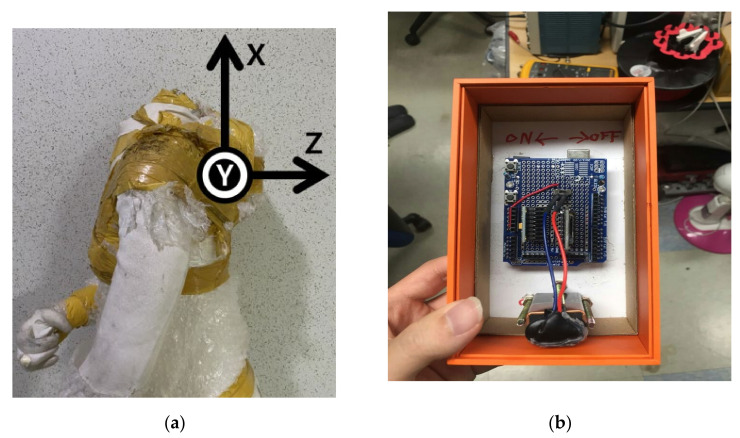
Three-axis of MPU 6050: (**a**) X, Y, Z axes indicated the head, arm, and body directions; (**b**) MPU6050 with battery.

To know the wearer’s motion state, the accelerations and angular velocities were measured using an Arduino device and an MPU6050 sensor. Figure 2a,b shows that the sensor has X, Y, Z axes, each of which indicates the head, arm, and body directions, respectively. The acceleration and angular velocity values depending on the wearer’s motion state were measured using this sensor. The data values measured via the sensor were corrected for noise using the complementary filter and a moving-average filter. Since the MPU6050 sensor data value is unstable, and errors can accumulate, an appropriate correction is required. First, by using a moving average filter, the average value was obtained through the data bus of 10 columns, and after squaring and summing them, the root value was obtained. The sensor value was corrected by the difference between this value and the gravitational acceleration. A complementary filter was used along with a moving average filter. Complementary and Kalman filters are usually used extensively, but the complementary filter was chosen because the processing of the Kalman filter took longer than that of the complementary filter. To prevent accumulated errors due to the integral calculation, the weights of acceleration and angle data were varied and corrected according to the stopping and driving conditions [26,27,28,29,30,31]. When it is close to a stationary state, the weight of acceleration is increased, and when a sudden rotation or movement is detected, the weight of angular velocity is increased to respond to the changed angle.

The learning data were largely classified into 2 kinds of cases and collected. The data collected on Case1 were general motion states (including movement, e.g., stretching), and on Case2, wearer’s motion state amid occurrence of accidents; the accident situations were divided into the front, rear, left, right, and fall situations. Since there is a difference in characteristics of acceleration and angular velocity depending on each situation, the accident situation and non-accident situation were supposed to be determined through artificial intelligence learning by using this difference [32]. The experiments to collect data were conducted 20 times according to each situation, and the measurement period was 50 ms [26,27,28,29]. A total of about 658 data were collected, including non-accident data. If there are more data, it is advantageous for learning, but due to the characteristics of collecting accident data, it was impossible to collect more data due to risks and equipment destruction. The reason for measuring 50 ms was that the data were saved in real time through the process of sensor→Arduino→save csv file, and the period it took was slightly shorter than 50 ms. Therefore, for ease of data processing later, the experiment was conducted by setting it to 50 ms.

#### 2.1.2. Raw Data Analysis

**Figure 3:**
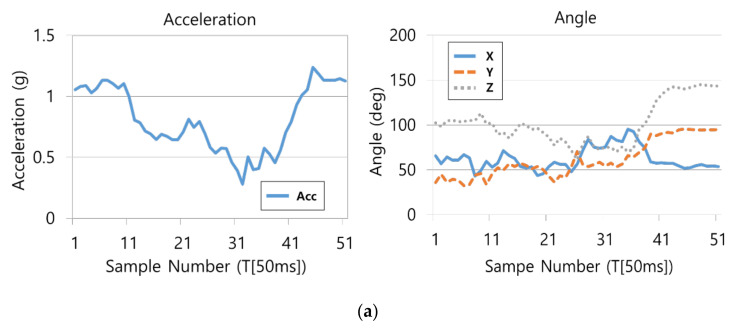

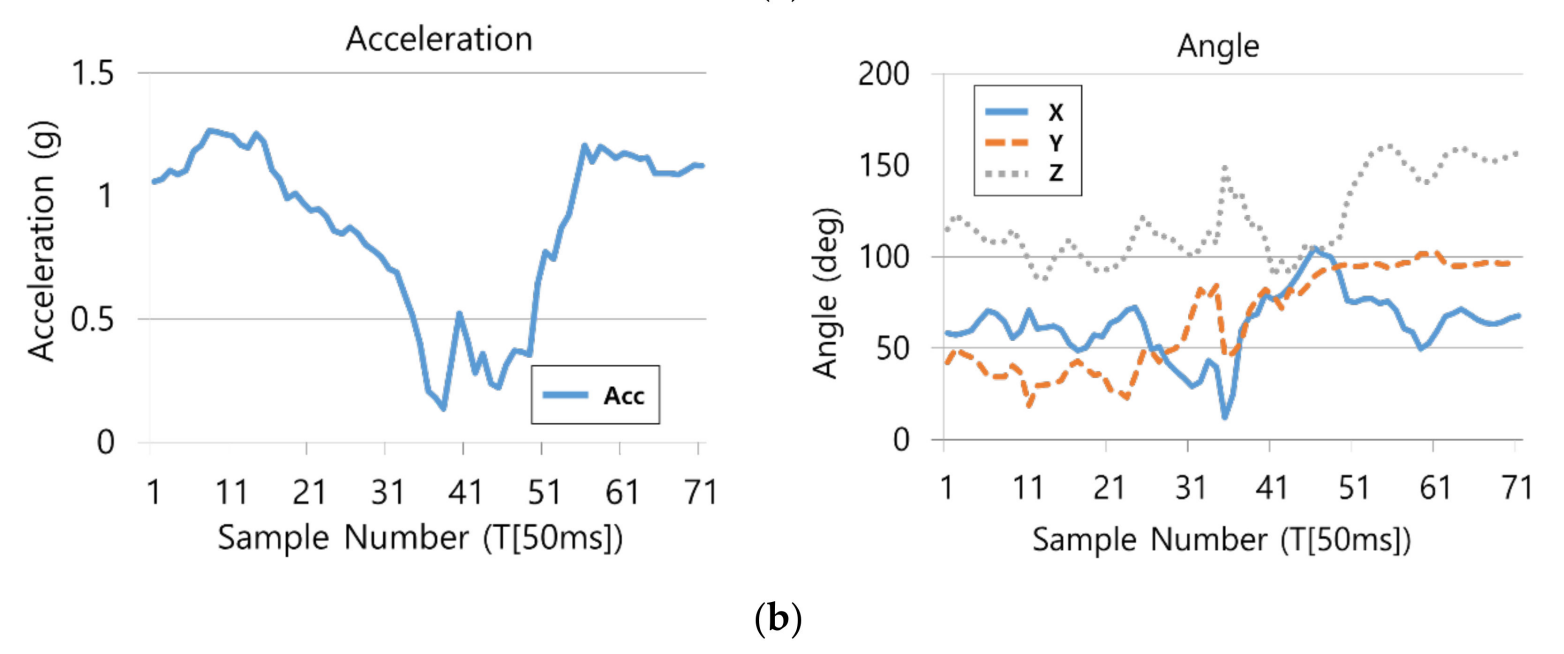
Similar graphs in the same accident situation(left collision): (**a**) accident 1; (**b**) accident 2.

The most significant difference between the accident situation and the non-accident situation is based on which one of the acceleration and angular velocity axes varies and how much it varies in a short time. Depending on the information derived from the posture type and the extent of the impact, the values indicated by the acceleration and angular velocity axes may differ, and a similar value or outcome appears in a similar situation, which can be also confirmed through the precedent studies [26,27,28,29,32]. Figure 3a,b shows similar graphs in the same accident situation (left collision). Acceleration graph is the magnitude of the sum vector of accelerations in the X, Y, and Z axes. It can be inferred that the acceleration graphs on the left of (a) and (b) and angle graphs X, Y, Z on the right show a similar flow in the same accident situation.

When there is no occurrence of accidents with a stable motion, there is no significant variation in acceleration and angle values; however, when an accident occurred that changed the driver’s motion state rapidly, there was a rapid variation in acceleration and 3-axis angles. 

Through the similarity and variation in graphs per time depending on situations, the accident or non-accident was distinguished, and the accelerations and angles of the 3 axes were used as the input data for the artificial intelligence model.

#### 2.1.3. System Drive Part

**Figure 4:**
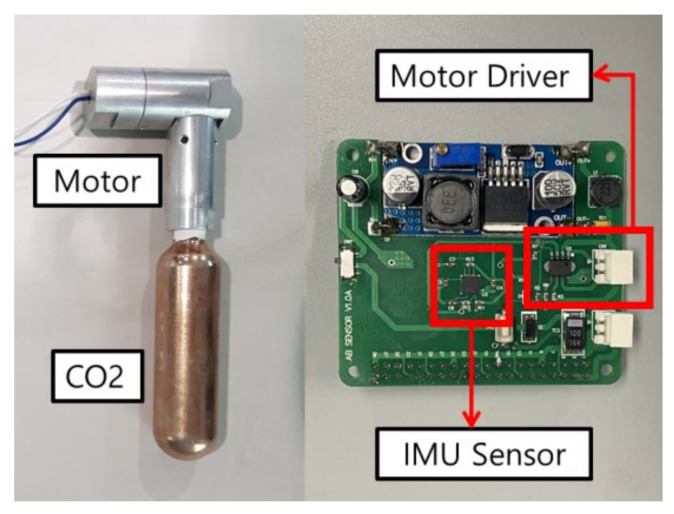
Measuring- and driving-part configuration.

Figure 4 shows the system drive part. The CO2 cartridge was used to inflate the airbags, and the DC motor was used to pop the CO2 cartridge. An algorithm is needed to send a signal to the DC motor, and in order to give a reference signal, a system that can be driven with artificial intelligence is needed. The Raspberry Pi 3B micro-computer was used for the artificial intelligence model, and the MPU6050 sensor was used to measure the driver’s motion status to determine the situation [26,27,28,29]. 

**Figure 5:**
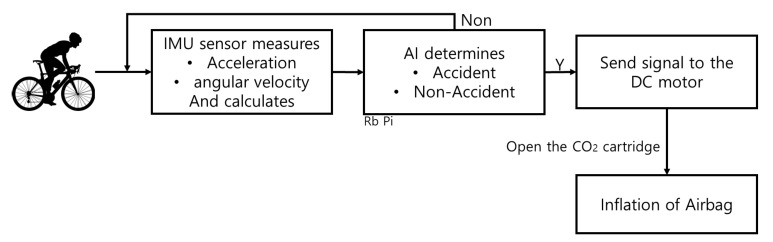
System diagram of the algorithm.

The driver’s motion state was measured using the MPU6050 to calculate the accelerations and angular velocities, and while the data file was stored separately, it was determined whether an accident had occurred through an artificial intelligence model. In the non-accident situation, the motions were continuously measured, and in the case of an accident, signals were given to the motor to inflate the airbag. Figure 5 shows a system diagram of the algorithm.

### 2.2. LSTM Theory and Design

**Figure 6:**
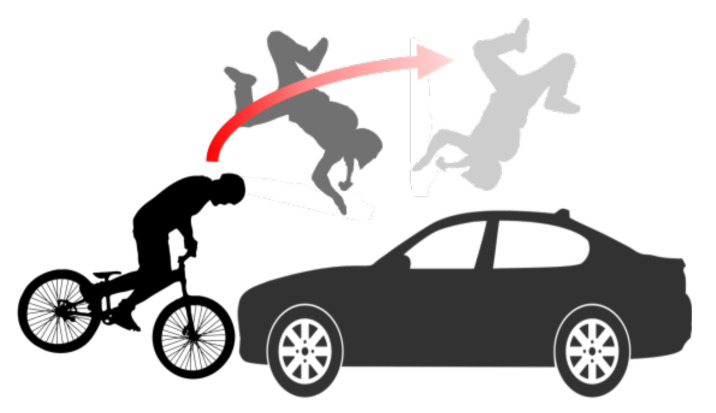
Sequential accident situation.

The NN, CNN, etc. used in the precedent studies produce outputs by making a judgment with respect to the currently inputted data. In these cases, the information on the data inputted before the currently inputted data is lost. The events of generating traffic accidents can be regarded as data sequence. The current motion of a rider after the occurrence of a collision is a result depending on the form of the previous motions, and therefore, it has a sequence such as that presented in Figure 6.

The RNN model can receive and utilize the past value. LSTM is a model that makes it possible to remember information from a long time ago by compensating for the inability to remember information far from the output, which is one of the features of RNN. This determines whether to forget, save, update, or output a piece of information through the sigmoid layer and the tanh layer. After passing through the sigmoid function, a value between 0 and 1 is outputted, which is the amount of information that has been deleted. The closer the value is to 0, the more the information is forgotten, and the closer it is to 1, the more remembered is the information. After passing through the tanh function, a value between –1 and 1 is outputted, deciding what information to store in the cell state. This paper used the LSTM model to recognize the accident situation, in which learning was carried out through the data on the accident situation. The LSTM model has a structure with a cell state added to the hidden state in the existing RNN algorithm and is composed of the input gate, cell state, forgetting gate, and output gate. 

**Figure 7:**
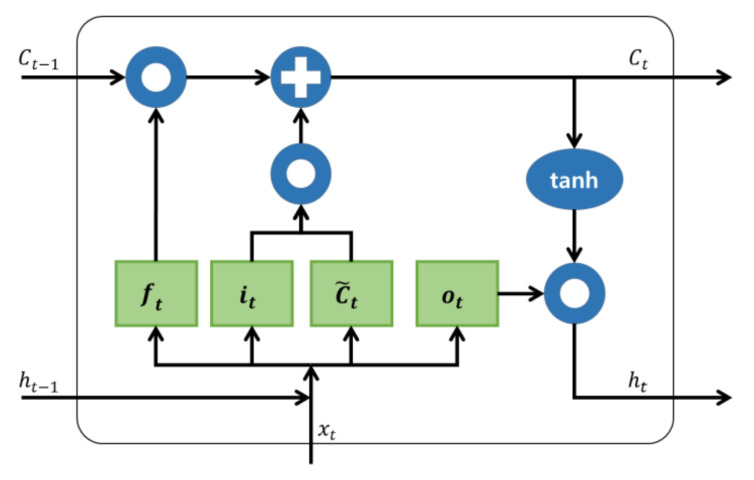
Cell operation process of LSTM.

Figure 7 shows the cell operation process of LSTM. The $f_{t}$ is the forgetting gate, $i_{t}$ is the input gate, ${\tilde{C}}_{t}$ is a generated vector of new values, $C_{t}$ is the cell gate, $o_{t}$ is the output gate, and $h_{t}$ is the output. *W* is weight at the gate, and b is the bias at the gate. σ is the sigmoid layer, tanh is the tanh layer, and ∘ is the Hadamard product [11,12,13,14]. The sigmoid and tanh layers are activation functions.

(1) $$f_{t}=\sigma \left(W_{f}\cdot \left[h_{t\;-\;1},x_{t}\right]+b_{f}\right),$$

(2) $$i_{t}=\sigma \left(W_{i}\cdot \left[h_{t-1},x_{t}\right]+b_{i}\right),$$

(3) $${\tilde{C}}_{t}=\tanh\left(W_{C}\cdot \left[h_{t-1},x_{t}\right]+b_{C}\right),$$

(4) $$C_{t}=f_{t}\circ C_{t-1}+i_{t}\circ {\tilde{C}}_{t},$$

(5) $$o_{t}=\sigma \left(W_{o}\cdot \left[h_{t-1},x_{t}\right]+b_{o}\right),$$

(6) $$h_{t}=o_{t}\circ \tanh\left(C_{t}\right)$$

**Figure 8:**
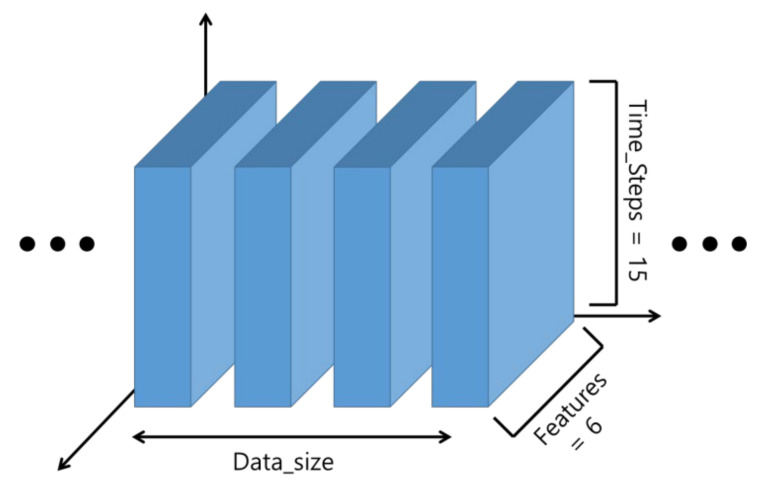
A three-dimensional array of LSTM’s input.

**Figure 9:**
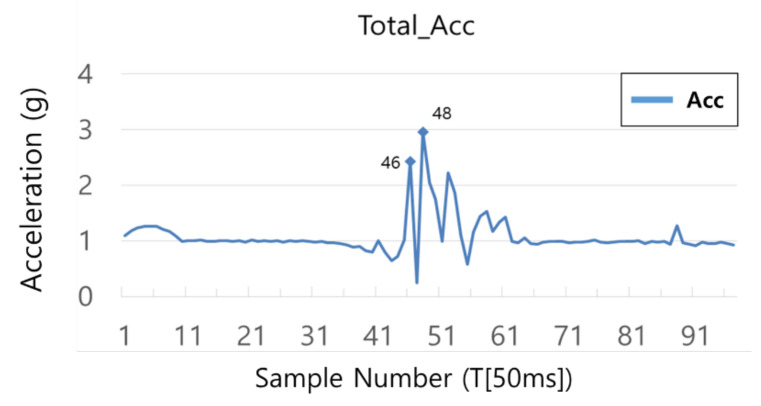
The accident occurred in the 46T–48T section.

The input of the LSTM model is a 3D array, as shown in Figure 8. The data used in this paper were obtained by correcting the 3 axes of acceleration and 3 axes of the angle measured by the MPU6050 sensor. In total, 15 sampling data of 6 axes, that is, 90 data were inputted. Table 1 shows a example of the input data. The input data were inputted, through an action similar to sliding while going over to the next data every 50 ms. When a person typically falls, it takes about 750 ms. It is 15 times the 50 ms, which was used as the sampling time of this system. Although 15 sets of data are inputted by axis based on 750 ms [33], in the bike accident, the falls may occur clearly at a faster speed [32], which can be confirmed from the collected data as well. In Figure 9, an accident occurred in the 46T–48T section and did not exceed 100 ms. The data during the pre-treatment process were cropped at the section before impacts were applied to the driver after the occurrence of the accident, and they were used as the input data.

Most of the acceleration values are between −2 g and 2 g, and the angle values are between −180 and 180 deg. The acceleration was divided by 2, and the angle was divided by 180 so that the values of the input data were almost between −1 and 1. 

**Figure 10:**
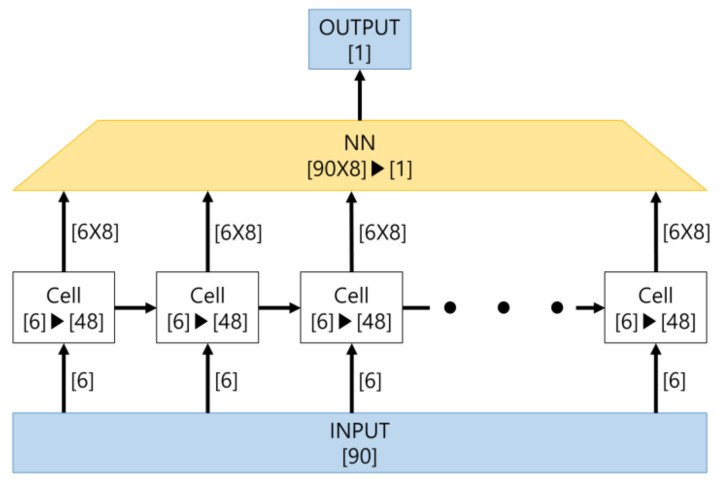
The structure of LSTM in this paper.

The number of cells was 15. Six input data were inputted per cell, and nodes amounted to 48. The output of LSTM was entered as the input of NN and finally outputted via NN. Figure 10 shows the structure of LSTM in this paper.

The NN received the output of LSTM as an input, proceeded through 4 hidden layers to reduce to 90, 30, 15, 8, and then obtained the final output. An activation function was used for each layer. The 1st, 3rd, and 4th hidden layers used tanh functions, and the 2nd hidden layer used ELU functions. The last output layer used sigmoid functions to classify the output into a binary. This was to distinguish between the accident and the non-accident by 1 and 0, respectively. Dropout was used to prevent overfitting. Cross entropy was used as a loss function and the adaptive moment estimation (ADAM) is used as an optimization algorithm to help learn inertially, but also adjustably according to the state of the parameters [34,35,36,37,38,39]. 

This study used the Jupyter notebook and Tensor Flow in algorithm design, and also designed the NN and CNN for comparison. The NN had the same structure as the NN used in the LSTM model. The CNN used the structure used in the precedent studies [20,21,22,23]. The convolution layer used 8 sets of 15 × 1 × 1 three-dimensional matrices as a kernel, and the stride was composed of a 1 × 1 × 1 matrix. The zero-padding technique was such that the size of the characteristic map became equal to the size of the input map, and the max pooling was used as the pooling operation.

## 3. LSTM Experiment Results and Comparisons

### 3.1. Test Results and Comparisons

For LSTM and CNN/NN, 1000 times of epochs were conducted, respectively. The size of the batch used in experiments was equal to that of the data used in learning, so weights were renewed once per epoch.

**Figure 11:**
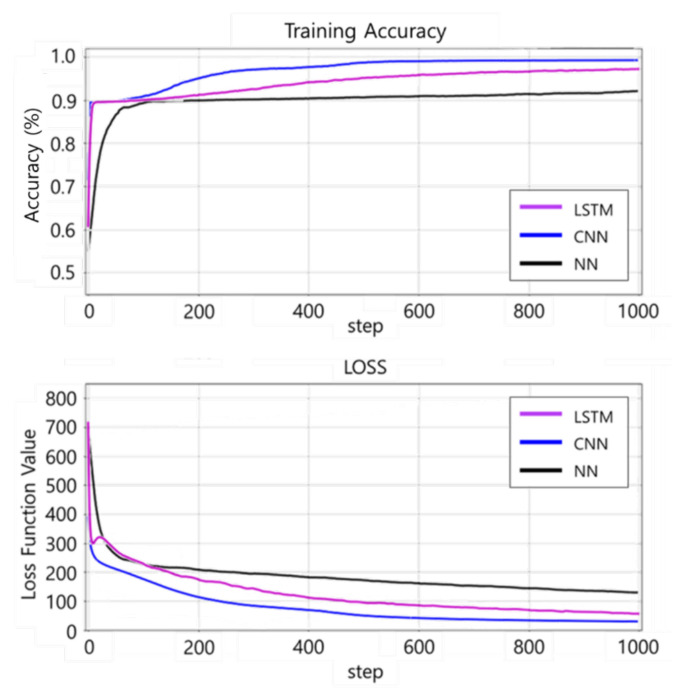
Training accuracy and loss. The horizontal axis of the graphs indicates the number of learning cycles.

Figure 11 shows the training accuracy and loss during 1000 times of epochs for LSTM and CNN/NN. The training accuracy indicates how many pieces of data inputted during the 1000 times of learning gave the correct answers. In terms of training accuracy, the CNN (99%) was the highest, followed by the LSTM (97%) and NN (92%). In terms of loss, the CNN (30) was the lowest, followed by the LSTM (55) and NN (128). To evaluate the test performance, the pre-separated test data were entered and checked. When training the artificial intelligence, the accident and non-accident data separated for testing, except for learning, were used, and the levels of accuracy of the neural networks were checked through testing. This is called test accuracy.

Tests were conducted 50 times, and Table 2 shows the assessments made by NN, CNN, or LSTM with respect to the non-accident and accident data. The graphs on the left represent the accelerations and angles with respect to non-accident and accident data, and the standards of the correct answer are 0 for non-accident and 1 for accident data. 

For non-accident data, the correct answer approaching 0 means that a correct judgment was made. Although all of NN, CNN, and LSTM outputted values near 0, the LSTM model was closest to 0. 

For accident data, the correct answer approaching 1 means that a correct judgment was made. The NN outputted a value of 0.734 once among two accidents but outputted a value of 0.205 for the remaining one time, making a wrong judgment. The CNN made a superior judgment to NN, but the LSTM model showed a point of 0.9, indicating the highest accuracy.

Table 3 represents a table of average values obtained 50 times of experiments, respectively, for NN, CNN, and LSTM with respect to training accuracy, test accuracy, training time, and test time. σ is the standard deviation. For training accuracy, the CNN showed the highest value of 98.87%, followed by the LSTM and NN models. The training time is the average time taken for the artificial intelligence to optimize up to the parameters through the output after receiving the input, and the test time is the time taken for the data to be inputted into the trained artificial intelligence model and then outputted. For the training time, the NN had the fastest time of 22.54 ms, and the LSTM had the longest time. When the data were inputted and tested after training, the LSTM showed the best test accuracy of 98.25%, compared to the training accuracy. For the test time, the NN took the shortest time, 1.34 ms, as it did for the training time. A fast test time means that the data judgment occurs rapidly when used in an actual situation. Of course, the LSTM model had a 0.5 ms (=0.0005 s) difference from the NN. However, the sampling time was 50 ms (=0.05 s), and the test time was very small, compared to the sampling time, and thus negligible. The training accuracy was higher in CNN than in LSTM, but the test accuracy was higher in LSTM. This means that when the CNN trains, there is a possibility of mistaking the feature points for the training data. When CNN recognizes a graph from an image point of view, it is possible to misjudge a non-accidental motion of a similar shape as an accident. Since LSTM checks the changing process of the axis itself, it can be analyzed with more focus on the change of the axis.

**Figure 12:**
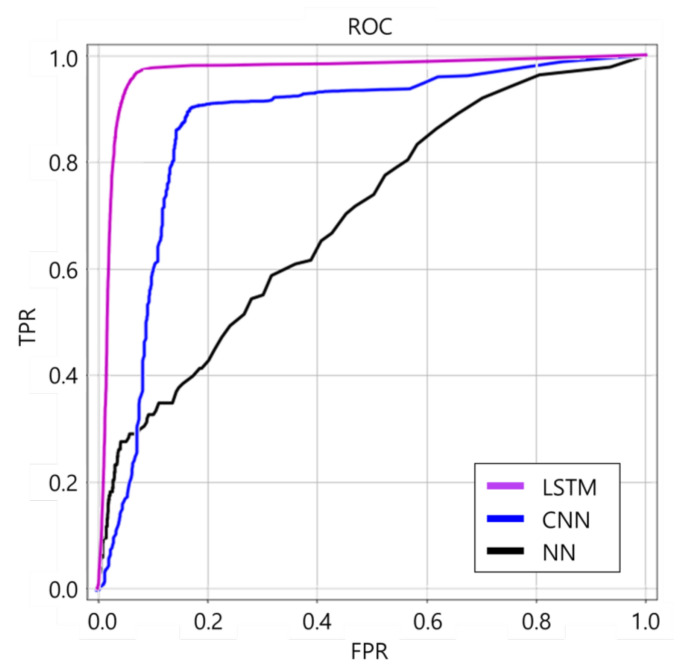
ROC curves of LSTM, CNN, and NN.

Figure 12 shows the receiver operating characteristic (ROC) curves of LSTM, CNN, and NN. The ROC curve is a curve representing the performance of how well the class of binary classification can be distinguished [22]. For example, when an accident situation is set to 1, and a non-accident situation is set to 0, there are four cases: 1 is judged as 1 (true positive), 0 is judged as 1 (false positive), 1 is judged as 0 (false negative), and 0 is judged as 0 (true negative). Additionally, through these classifications, sensitivity, specificity, accuracy, error rate, precision, etc. can be identified. The *x*-axis (false positive rate (FPR)) shows a case that non-accident (0) is judged as an accident (1), and the *y*-axis (true positive rate (TPR)) shows a case that an accident (1) is judged as an accident (1). It is possible to know what artificial neural network can distinguish between the accident and non-accident situations better by using the ROC curve for the artificial neural network with outputs 1 and 0 for accident and non-accident. The better the distinction is, the more the ROC curve leans to the corner side at the left top. According to the graph, the ROC curve of the LSTM model leans toward the utmost top left. Next to the LSTM, the CNN model leans toward the top left, and the NN shows a lower distinguishment performance than the other two artificial neural network models.

### 3.2. Testing with Airbag

**Figure 13:**
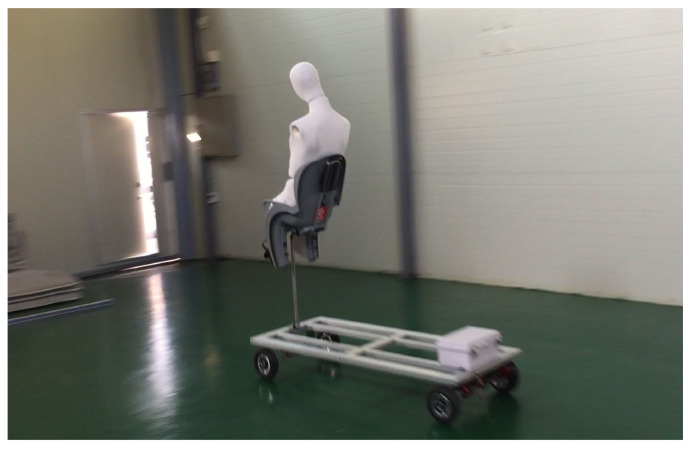
Motorcar with a mannequin for collision testing.

After completion of the artificial intelligence training and simulation testing, to test it by applying it to an actual hardware system, a collision situation was reproduced using a mannequin and a motorcar, as shown in Figure 13. 

**Figure 14:**
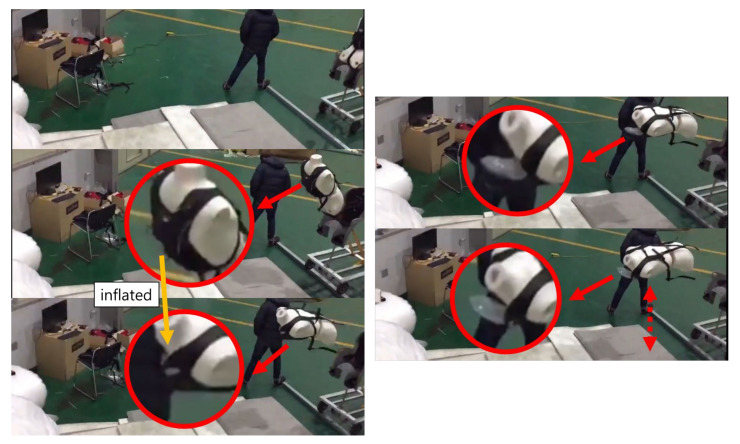
Airbag operation confirmation before the collision.

The LSTM program was inserted into the Raspberry PI, combined with the MPU6050 sensor module, etc., and it was made into a bag with an airbag and worn by a mannequin. When the motor car collides with an obstacle, and the mannequin flies off, it shows the airbag exploding from the bag the mannequin was wearing. It was confirmed that the airbag inflated before the mannequin hit other objects, as shown in Figure 14.

## 4. Conclusions

This paper proposed a system to operate an airbag by judging whether an accident involving a bike driver occurred using LSTM. When using the time series feature of the data using LSTM, it was confirmed that the accuracy of judgment was higher than that of a single judgment on the existing input data. Through these strengths, it could predict whether the current situation is an accident by using the time information on the change in the driving state of the driver after the occurrence of an accident. In addition, it shows it is possible to train a model using the sensor data with respect to three acceleration axes and three angle axes, and judge and distinguish the situation from these data. Thus, we confirmed the possibility of a system that can protect the rider by judging the accident situation of the rider using the IMU sensor and artificial intelligence algorithm, and operating the airbag according to the situation. 

In this study, the number of nodes and rarities of NN, CNN, and LSTM were limited to find an optimized model in the same environment, but to optimize the model itself, it is necessary to simplify the structure of the plant by reducing the weight or parameters of the model. To be sure, more data should be collected in order to judge other additional behaviors. If so, it is expected that it can be used for bikes as well as for other uses. However, there is an issue about the black box problem, i.e., the process of accident judgment is not yet known. Additional research such as explainable artificial intelligence (XAI) is needed to interpret the malfunction and the process of deriving the correct answer. Through such studies, the correlation between the given data and the answer inferred by the algorithm is analyzed, and it is expected that the reliability and stability of the model can be further increased.

## Figures and Tables

**Table 1:** Input data of the LSTM model used in this study.

Time	Acceleration			Angle		
	X	Y	Z	X	Y	Z
T_s1	1.08873	0.07507	−0.12464	18.58213	89.26502	66.49303
T_s2	0.93897	0.02329	0.53261	25.70627	88.88076	64.56924
T_s3	0.96653	0.08587	0.65714	32.06958	86.69562	59.52053
T_s4	1.23658	−0.25690	0.09096	23.78130	92.49972	66.96293
T_s5	0.53428	0.02383	0.28870	23.87306	91.96020	66.31092
T_s6	0.89975	0.44901	0.57337	26.97208	87.61472	64.80787
T_s7	0.68992	0.22347	0.56436	32.90183	83.46288	57.45921
T_s8	0.64561	−0.21184	0.76713	45.35566	89.55425	49.63304
T_s9	1.10645	−0.63056	0.31480	42.66323	96.83949	55.33480
T_s10	0.94762	−0.25137	0.87484	43.45735	98.79564	52.71785
T_s11	0.63805	−0.30686	0.60364	44.29453	103.07350	52.60575
T_s12	0.47248	−0.27727	0.46273	46.30504	109.24230	51.72902
T_s13	−1.23561	−0.02847	0.10609	64.33788	96.78557	66.50202
T_s14	1.07818	−0.02991	−0.45904	58.99714	93.76888	80.94142
T_s15	1.28572	−1.29456	−1.54209	78.00906	113.27850	84.13935

**Table 2:** Comparison of a NN, CNN, and LSTM.

	Acceleration	Angle	Correct Answer	NN	CNN	LSTM
No Accident	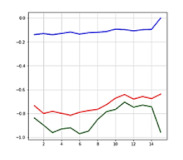	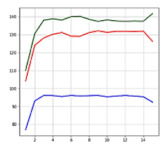	0	0.189	0.038	0.006
No Accident	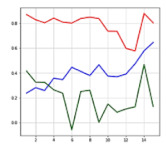	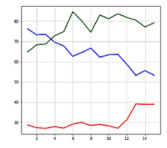	0	0.194	0.033	0.002
Accident	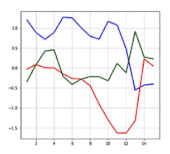	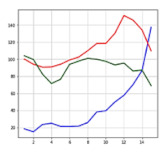	1	0.734	0.954	0.932
Accident	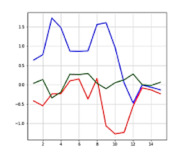	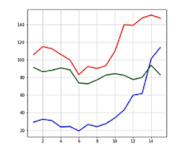	1	0.205	0.758	0.943

**Table 3:** Comparison of training and test results by type of artificial intelligence algorithm.

	NN	CNN	LSTM
**Tr_Acc[%] (σ)**	91.96 (1.23)	98.87 (1.38)	97.17 (0.50)
**Ts_Acc[%] (σ)**	86.75 (4.78)	95.75 (3.41)	98.25 (3.54)
**Tr_Per[ms] (σ)**	22.54 (1.18)	31.87 (1.36)	41.83 (2.36)
**Ts_Per[ms] (σ)**	1.34 (0.47)	1.50 (0.50)	1.88 (0.48)
